# Characterization of the Complete Genome Sequence of a Beak and Feather Disease Virus from a Moluccan Red Lory (*Eos bornea*)

**DOI:** 10.1128/genomeA.00844-13

**Published:** 2013-11-27

**Authors:** Subir Sarker, Seyed A. Ghorashi, Jade K. Forwood, Stewart Metz, Shane R. Raidal

**Affiliations:** School of Animal and Veterinary Sciences, Charles Sturt University, Wagga Wagga, New South Wales, Australiaa; School of Biomedical Sciences, Charles Sturt University, Wagga Wagga, New South Wales, Australiab; Graham Centre for Agricultural Innovation, Wagga Wagga, New South Wales, Australiac; Kembali Bebas, Masihulan, Seram, Indonesiad; Indonesian Parrot Project, Sebastapol, California, USAe

## Abstract

The complete genome sequence of a beak and feather disease virus (BFDV) encoding two major open reading frames (ORFs) was characterized in a wild Moluccan red lory (*Eos bornea*). This is the first report of a BFDV genome from Indonesia and the first reported BFDV infection for this host species.

## GENOME ANNOUNCEMENT

Beak and feather disease virus, the etiologic agent of psittacine beak and feather disease (PBFD), is a nonenveloped icosahedral virus (~20 mm in diameter) with an approximately 2.0-kb circular single-stranded DNA (ssDNA) genome encoding two bidirectionally transcribed major open reading frames (ORFs) (1–3). It is one of the potentially fatal infections affecting more than a quarter of all known parrot species globally, which are considered to be on the edge of extinction (4). Infection by beak and feather disease virus (BFDV) leads to long-term immunosuppression, massive viral excretion, and enduring antibody-negative status (5). The disease can be expressed acutely in juvenile birds, with a presentation ranging from sudden death to a chronic progressive form that is often characterized by symmetrical feather abnormalities, and in some cases in beak and claw deformities (6, 7). Here, we report the molecular characterization of a BFDV genome from a Moluccan red lory (*Eos bornea*) in the wild of Seram Island, Indonesia.

The BFDV viral genomes were amplified from dried blood spots collected from *Eos bornea* (sample ID 05-1174) (year of sampling, 2005; GPS location, -2.978699°S 129.222865°E) and the genomic DNA was extracted using established protocols (8–10). To amplify the entire viral genome, a published primer (BFDV-P2, 5′-AACCCTACAGACGGCGAG-3′) (8) and designed primers (BFDV-J-R, 5′-TTGGGTCCTCCTTGTAGTGG-3′; BFDV-I-F, 5′-GCAAACTGACGGAATTGAACATA-3′; and BFDV-C-R, 5′-CGTCCAACGATGGCATAGT-3′) were used. The reactions for the different primer sets were optimized, and the optimized reaction mixture contained 3 µl extracted genomic DNA, 2.5 µl of 10× High Fidelity PCR buffer (Invitrogen), 1 µl of 25 µM each primer, 1 µl of 50 mM MgSO_4_, 4 µl of 1.25 mM each deoxynucleoside triphosphate (dNTP), 1 U Platinum *Taq* DNA polymerase High Fidelity (Invitrogen), and distilled water (dH_2_O) added for a final volume of 25 µl. The optimized PCR conditions were as follows: 95°C for 3 min, followed by 40 cycles of 95°C for 30 s, 57°C for 45 s, and 68°C for 2 min, and finally 68°C for 5 min. The extension time for the second set of primers (BFDV-I-F and BFDV-C-R) was 1.5 min instead of 2 min. The amplified PCR products were TA cloned into pGEM-T vector (Promega) and sequenced at the Australian Genome Research Facility (AGRF) Ltd. (Brisbane, Australia). The sequenced contigs were assembled, and the entire BFDV genome was constructed using the Geneious software (version 6.1.6).

The complete genome sequence of BFDV (GenBank accession no. KF673337) comprised 2,002 nucleotides (nt), with a G+C content of 54.6%, and ORF1 and ORF2 encode 975 nt and 744 nt, respectively. A phylogenetic analysis of this Moluccan red lory genome with all other BFDV genomes available on GenBank revealed the closest relationship (100% bootstrap support and >90% nucleotide sequence identity) with one Australian BFDV genome (GenBank accession no. AF311299). This is the first report of a BFDV genome from Indonesia and the first reported BFDV infection for this host species.

### Nucleotide sequence accession number.

The complete genome sequence of BFDV has been deposited at GenBank under the accession no. KF673337.
